# Exploring the Application Potential of Aquaculture Sewage Treatment of *Pseudomonas chengduensis* Strain WD211 Based on Its Complete Genome

**DOI:** 10.3390/genes14122107

**Published:** 2023-11-21

**Authors:** Huanlong Peng, Hangtao Wu, Wenjie Gu, Yusheng Lu, Hongjie Qin, Yi You, Donglai Zhou, Dan Wang, Lili Sun, Changmin Zhou, Yanling Zheng

**Affiliations:** 1 Institute of Agricultural Resources and Environment, Guangdong Academy of Agricultural Sciences, Key Laboratory of Plant Nutrition and Fertilizer in South Region, Ministry of Agriculture, Key Laboratory of Nutrient Cycling and Farmland Conservation of Guangdong Province, Guangzhou 510640, China; 2 Maoming Branch, Guangdong Laboratory for Lingnan Modern Agriculture, Maoming 525000, China; 3 Environmental Horticulture Research Institute, Guangdong Academy of Agricultural Sciences, Guangzhou 510640, China; 4 Sericultural & Agri-Food Research Institute, Guangdong Academy of Agricultural Sciences, Guangzhou 510610, China

**Keywords:** *Pseudomonas chengduensis*, complete genome, purification capability, antibiotic resistance, aquaculture sewage

## Abstract

*Pseudomonas chengduensis* is a new species of *Pseudomonas* discovered in 2014, and currently, there is a scarcity of research on this bacterium. The *P*. *chengduensis strain WD211* was isolated from a fish pond. This study investigated the purification capability and environmental adaptability of strain WD211 in wastewater and described the basic features and functional genes of its complete genome. According to the results, the sewage treated with strain WD211 showed a decrease in concentration of 18.12% in total nitrogen, 89.39% in NH_4_^+^, 62.16% in NO_3_^−^, 79.97% in total phosphorus, and 71.41% in COD after 24 h. Strain WD211 is able to survive in a pH range of 6–11. It shows resistance to 7% sodium chloride and different types of antibiotics. Genomic analysis showed that strain WD211 may remove nitrogen and phosphorus through the metabolic pathway of nitrogen assimilation and phosphorus accumulation, and that it can promote organic decomposition through oxygenase. Strain WD211 possesses genes for producing betaine, trehalose, and sodium ion transport, which provide it with salt tolerance. It also has genes for antibiotic efflux and multiple oxidases, which give it antibiotic resistance. This study contributes to the understanding of the sewage treatment ability and potential applications of *P. chengduensis*.

## 1. Introduction

With the advancement of the aquatic artificial culture industry, there has been an increase in the use of aquatic feeds, leading to the release of a significant amount of nitrogen, phosphorus, and other elements into water [1,2,3]. Excessive nitrogen and phosphorus can lead to the eutrophication of water, while excessive nitrite and ammonia nitrogen can be toxic to animals [4]. Moreover, the use of various drugs, such as antibiotics, during the breeding process further contributes to water pollution [5]. Biological methods have become competitive methods for removing water pollutants due to their economic feasibility, high efficiency, and environmental friendliness [6,7].

In aquaculture, farmers commonly use a range of antibiotics, such as tetracycline, sulfonamides, aminoglycosides, and quinolones, to prevent and control animal diseases [8]. These antibiotics can cause a significant decrease in the performance of the biological treatment of sewage [9]. In addition, there may be other organic compounds, salts, and other substances in sewage that are toxic to functional microorganisms and reduce the effect of water purification. Therefore, when screening microorganisms for nitrogen and phosphorus removal, attention also needs to be paid to whether the strains can tolerate the complex environment in wastewater.

*Pseudomonas* is a group of Gram-negative bacteria, which is one of the most numerous and complex genera of bacteria known [10]. Microorganisms in this genus have extremely rich metabolic and functional diversity, which allows them to adapt to a wide range of environments and survive in a very wide range of ecological niches [11]. It has been found that functional differences in *Pseudomonas* depend on their ecological niche and that strains from specific environments possess a unique set of metabolic pathways and functions [12]. Since many genes are silenced under laboratory conditions, complete genome sequencing can help researchers to gain a more comprehensive understanding of microbial metabolism and function [13]. *Pseudomonas chengduensis* is a species of *Pseudomonas* that was newly discovered in 2014 [14], for which there is a paucity of research. Genes related to polyethylene terephthalate (PET) degradation, cadmium tolerance, and denitrification in the strain have been described from its genome [15,16,17], of which only the description of PET degradation genes is based on the complete genome. Important genomic insights into the aquaculture sewage treatment of this species remain undescribed.

In this study, we tested the sewage treatment capacity (nitrogen and phosphorus removal, organic matter degradation) and abiotic stress tolerance (salt, pH, antibiotic) of *P. chengduensis* WD211 isolated from a fish pond. At the same time, the complete genome of the strain was sequenced and analysed, the basic characteristics of the genome were described, and the sewage treatment and resistance ability of *P. chengduensis* was discussed at the complete-genome level, which provided a basis for genetic and functional research on *P. chengduensis*.

## 2. Materials and Methods

### 2.1. Sewage Treatment Capacity

*P. chengduensis* WD211 is preserved as No: CGMCC No. 23,926 in the China General Microbiological Culture Collection Center (CGMCC). Strain WD211 was grown in NB medium (5 g/L of beef extract, 10 g/L of peptone, and 5 g/L of sodium chloride) at 30 °C and shaken at 200 rpm for 24 h. After centrifugation, the bacterial suspensions were adjusted to an OD_600_ of 1.0 with 0.9% sodium chloride solution. The bacterial suspensions in 1.5 mL volumes were inoculated in 150 mL of fish pond wastewater and shaken at 30 °C for 96 h at 200 rpm (in a 250 mL triangular flask), and samples were collected every 24 h. Samples were filtered through a 0.22 μm microporous membrane and tested for ammonia nitrogen, nitrate nitrogen, total nitrogen, COD, and total phosphorus according to standard methods [18]. Fish-pond water without bacterial solution was used as a control. Every experiment was conducted with three replicates, and we took the average value.

The pH of the fish-pond wastewater was 7.6. The total nitrogen, NH_4_^+^, NO_3_^−^, total phosphorus, and COD concentrations of the fish-pond wastewater were 19.17, 3.61, 3.81, 0.99, and 144.2 mg/L, respectively.

### 2.2. Antibiotic Susceptibility Tests

Strain WD211 was incubated for 24 h, centrifuged, and resuspended in fresh NB medium, and the OD_600_ absorbance of the bacterial suspension was adjusted to 1.0. The bacterial suspension was spread on NA (5 g/L of beef extract, 10 g/L of peptone, 5 g/L of sodium chloride, and 15 g/L of agar, pH 7.0) medium. Test strip sheets with antibiotics were placed in the centre of the Petri dish. The Petri dishes were incubated at 30 °C for 48 h and examined for circles of inhibition. The test strips with antibiotics were purchased from Hangzhou Microbiological Reagents Co., Ltd., Hangzhou, China.

### 2.3. pH and Salt Tolerance

Strain WD211 was incubated in NB medium for 24 h, centrifuged, and resuspended in fresh NB medium, and the OD_600_ absorbance of the bacterial suspension was adjusted to 1.0. The bacterial suspension was inoculated at 1% inoculum into NB medium containing different pH values (2, 3, 4, 5, 6, 7, 8, 9, 10, 11, and 12) and incubated at 30 °C. After 24 h and 48 h of incubation, growth was confirmed by measuring the OD at 600 nm. Every experiment was conducted with three replicates.

Strain WD211 was incubated in NB medium for 24 h, centrifuged, and resuspended in fresh NB medium, and the OD_600_ absorbance of the bacterial suspension was adjusted to 1.0. The bacterial suspension was inoculated at 1% inoculum into NB medium containing different NaCl concentrations (1%, 3%, 5%, 7%, 9%, and 11%) and incubated at 30 °C. After 24 h and 48 h of incubation, growth was confirmed by measuring the OD at 600 nm. Every experiment was conducted with three replicates.

### 2.4. Genome Sequencing and Annotation

The strain genome was sequenced with Beijing Biomarker using Illumina and OXfoad Nanopore platforms. Filtered reads were assembled using Canu v1.5 software; the assembly results were corrected with Racon v3.4.3 software using three generations of reads, cyclization and adjustment of start sites were performed using Circlator v1.5.5 software, and Pilon v1.22 software was used to further correct errors using the second-generation data to obtain a more accurate genome for subsequent analysis.

For genome component prediction, coding gene prediction was performed using Prodigal v2.6.3. The GenBlastA v1.0.4 program was used to scan the complete genomes after masking predicted the functional genes. Putative candidates were then analysed by searching for nonmature mutations and frame-shift mutations using GeneWise v2.2.0. Transfer RNA (tRNA) genes were predicted with tRNAscan-SE v2.0, and ribosome RNA (rRNA) genes were predicted with Infernal v1.1.3. Repetitive sequences were predicted using RepeatMasker. PhiSpy v2.3 was used for prophage prediction, and CRT v1.2 was used for CRISPR identification. IslandPath-DIMOB v0.2 was used to predict genomic islands in the genome. PromPredict v1 was used for promoter prediction. For functional annotation, the predicted proteins were blasted (e-value: 1 × 10^−5^) against Nr, Swiss-Prot, TrEMBL, KEGG, and eggNOG. Blast2go was used for GO annotation.

### 2.5. Whole-Genome Sequence Alignments

Annotated genomic sequences were aligned using progressive Mauve [19]. Local collinear blocks (LCBs) were derived using the Mauve default settings, and minimal LCB weights are reported. The genome accessions of *P. chengduensis* WD211, BC1815, and T1624 were NZ_CP129400.1, NZ_CP111110.1, and NZ_CP095766.1, respectively.

## 3. Results

### 3.1. Effect of Strain WD211 on Removing Pollutants from Fish-Pond Aquaculture Wastewater

**Figure 1:**
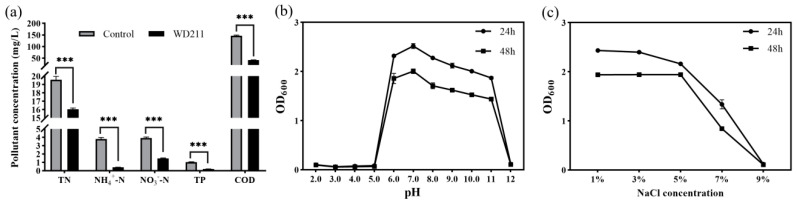
Test of the purification capacity and stress resistance of strain WD211. (**a**) The best effect was selected at 24 h. (**b**) Growth of strain WD211 at different pH values. (**c**) Growth of strain WD211 at different NaCl concentrations. *** *p* < 0.001 represents a significant difference.

Our results showed that strain WD211 can improve the quality of aquaculture wastewater (Figure 1a). The effluent inoculated with strain WD211 showed a decrease of 18.12% in total nitrogen, 89.39% in NH_4_^+^-N, 62.16% in NO_3_^−^-N, 79.97% in total phosphorus, and 71.41% in COD after 24 h. Strain WD211 has the potential to be applied for the treatment of a wide range of pollutants in aquaculture sewage. Strain WD211 has the best ability to remove NH_4_^+^-N, followed by total phosphorus and COD, and finally NO_3_^−^-N. It is noteworthy that strains with simultaneous removal of NH_4_^+^-N, NO_3_^−^-N, total phosphorus, and COD are rarely reported. 

### 3.2. pH and Salt Tolerance Ranges of Strain WD211

Strain WD211 was tested for its resistance, and the results are shown in Figure 1b. Strain WD211 was able to grow in the range of pH 6~11, the growth reached a maximum at pH 7 in the 24th hour, and the OD_600_ absorbance value reached 2.52. The growth of strain WD211 decreased slowly with the increasing pH, and the OD_600_ absorbance value still reached 1.87 at pH 11, which showed good alkali tolerance. The pH tolerance range of strain WD211 covers a suitable pH range for almost all types of aquaculture animals, making it suitable for use in the treatment of aquaculture sewage.

The salt tolerance test results showed that strain WD211 was able to tolerate up to 7% NaCl (Figure 1c). The OD_600_ absorbance value reached 2.43 after 24 h of incubation in medium with 1% NaCl, the growth gradually decreased with the increasing NaCl concentration, and the OD_600_ absorbance value decreased to 1.33 after 24 h of incubation in medium containing 7% NaCl. In medium containing 9% NaCl, growth was stopped.

### 3.3. Antibiotic Susceptibility of Strain WD211

Antibiotic tolerance test results showed that strain WD211 was able to tolerate many antibiotics (Table 1), and its growth was not affected by penicillin, carbenicillin, cefadroxil, cefazolin, cefradine, ceftazidime, ceftriaxone, cefoperazone, gentamycin, kanamycin, neomycin, tetracycline, doxycycline, minocycline, erythromycin, madecassicin, vancomycin, or cotrimoxazole. The growth inhibition zones of 18 tested antibiotics were 0 mm. WD211 was able to tolerate certain concentrations of benzoxacillin, ampicillin, cefuroxime, furazolidone, chloramphenicol, clindamycin, norfloxacin, and ciprofloxacin and was sensitive to piperacillin, butylcarbamazine, and ofloxacin.

### 3.4. General Features of Genome

**Figure 2:**
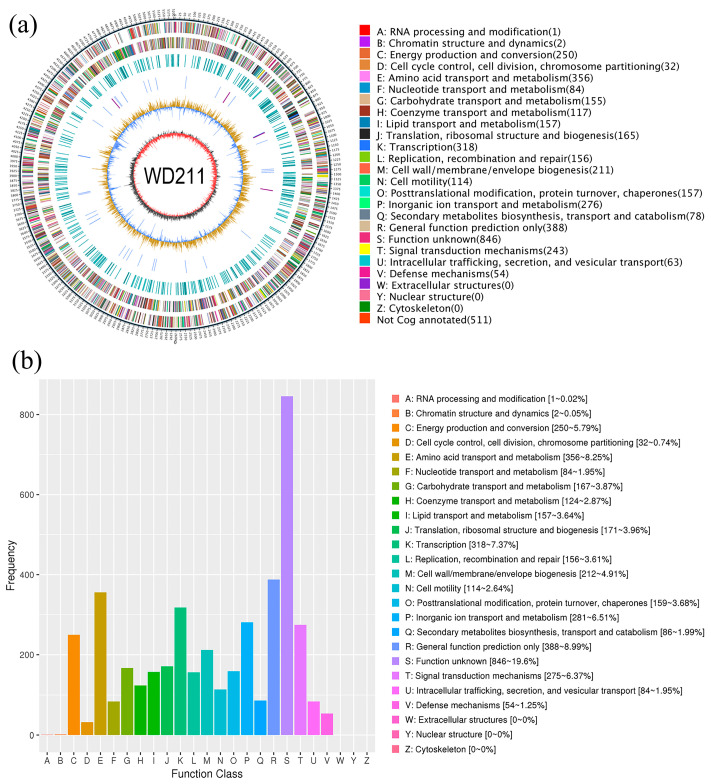
General features of the genome of strain WD211. (**a**) Circular chromosome map of strain WD211. (**b**) COG analysis of WD211.

The final assembled genome was a single circular chromosome (5,208,067 bp) without plasmids or gaps (Figure 2a). The outermost circle was a marker of genome size, with a scale of 5 KB each; the second and third circles were genes on the positive and negative chains of the genome, respectively; and different colours represent different COG functional classifications. The fourth circle is a repeating sequence; the fifth circle is tRNA and rRNA; the blue circle is tRNA; and the purple circle is rRNA. The sixth circle shows the GC content. The light-yellow part indicates that the GC content in this region is higher than the average GC content in the genome, and the higher the peak value, the greater the difference between it and the average GC content. The blue part indicates that the GC content in this region is lower than the average GC content in the genome. The innermost circle is GC-skew. The dark grey represents the area where the G content is greater than C, and red represents the area where the C content is greater than G. 

**Figure 3:**
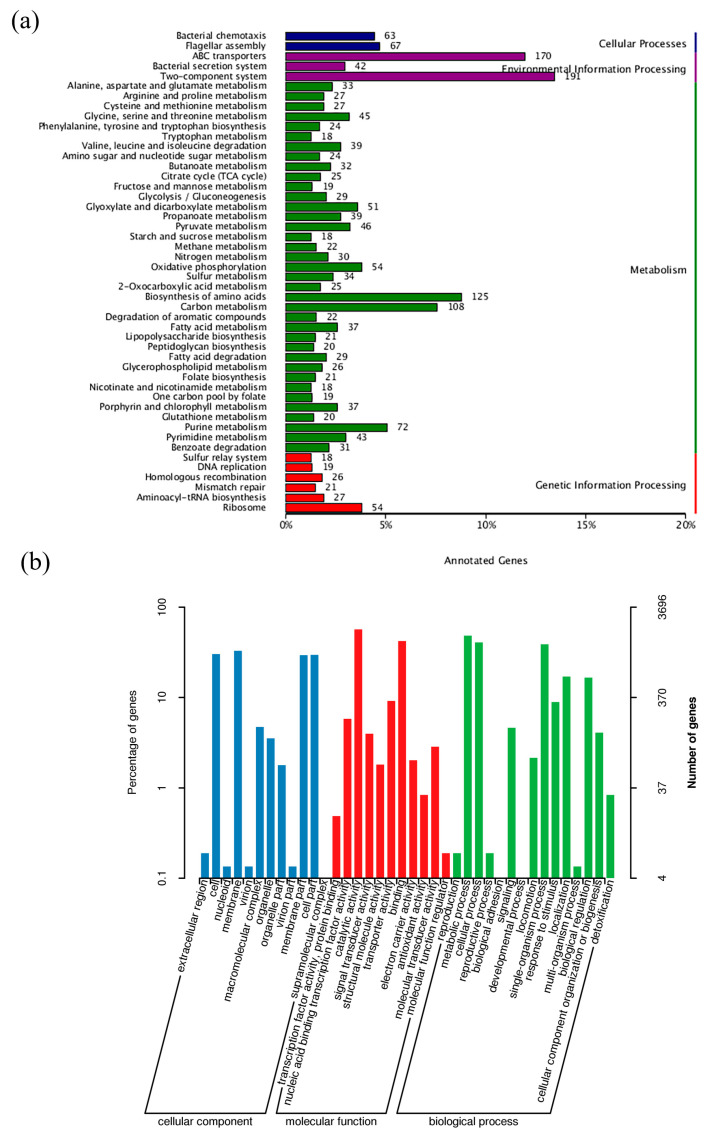
General features of the genome of strain WD211. (**a**) KEGG analysis of WD211. (**b**) GO analysis of WD211.

The G + C content was 62.75%, consisting of 4734 coding genes, 12 rRNA genes, and 67 tRNA genes. The genome of strain WD211 contained one pseudogene, two CRISPR, and 14 genomic islands (Table 2). A total of 4317 genes that were annotated according to the COG analysis were divided into 25 functional groups (Figure 2b); 2008 genes that were annotated according to the KEGG analysis were divided into five categories (Figure 3a) and 50 functional groups; and 3696 genes that were annotated according to GO were divided into three categories and 38 functional groups (Figure 3b).

**Figure 4:**
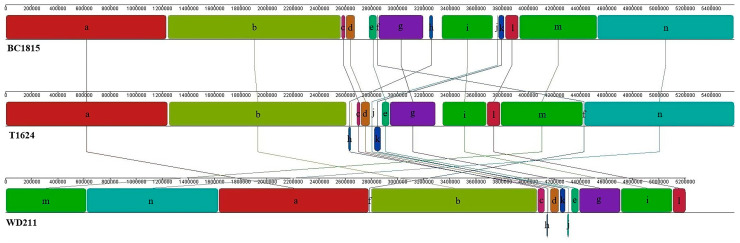
Whole-genome sequence alignments.

Currently, only two complete genomes of *P. chengduensis* have been made public, and only one of them has been briefly described [15]. We compared the genomes of two strains with that of a third strain, WD211, as illustrated in Figure 4. A total of 14 local collinear blocks (LCBs) were observed (minimum weight (MW)  =  6770) in all three strains. The genomic structures of the three strains were found to be significantly different. The genome of strain WD211 underwent gene rearrangements in fragments f, h, j, and k in contrast with the genome of BC1815. Similarly, the genome of WD211 underwent gene rearrangements in fragments c, f, j, and k in contrast with the genome of T1624. Furthermore, gene fragments h and i in the WD211 genome underwent gene inversions. These structural changes in the genome provide the WD211 strain with an advantage for acquiring new traits.

### 3.5. Genomic Insights into Sewage Treatment

#### 3.5.1. Genes Related to Nitrogen and Phosphorus Removal

According to the analysis of the genome, various nitrogen metabolism-related genes have been predicted in the genome of strain WD211. These genes play an important role in the nitrogen removal process of strain WD211. Among them, *nrt* and *nrtABCD* encode proteins responsible for transporting extracellular NO_3_^−^ and NO_2_^−^ into the cell. Specifically, they are involved in the nitrate/nitrite transport system, including the substrate-binding protein, permease protein, and ATP-binding protein. NO_3_^−^ is catalysed to NO_2_^−^ by the assimilatory nitrate reductase catalytic subunit and the assimilatory nitrate reductase electron transfer subunit, which are encoded by *nasA* and *nasB,* respectively. NO_2_^−^ is then catalysed to NH_4_^+^ by nitrite reductase NADH large subunits and nitrite reductase NADH small subunits, which are encoded by *nirB* and *nirD*, respectively. Extracellular NH_4_^+^ is transported directly into cells via ammonium transporters. Afterwards, facilitated by the enzymes encoded by *glnA*, *gltB*, *GLT1*, *gudB*, and *gdhA*, NH_4_^+^-N is transformed into L-glutamine, which is then utilized by bacteria in glutamine metabolism.

**Figure 5:**
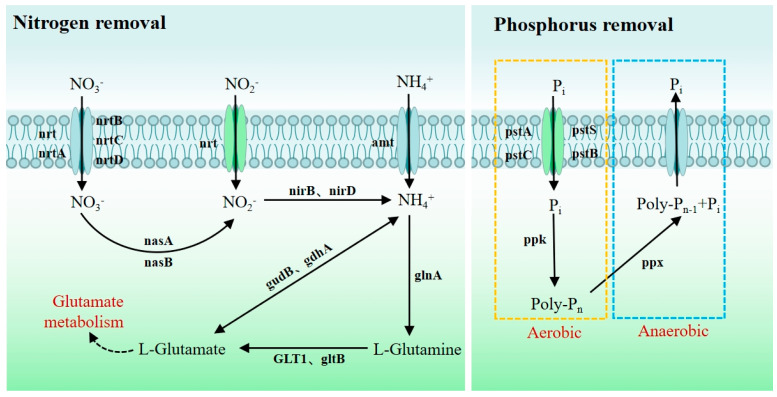
Metabolic pathways of nitrogen and phosphorus removal in strain WD211.

Furthermore, the analysis of phosphorus metabolism genes reveals that strain WD211 utilizes the phosphate transport system encoded by *pstABCS* to uptake phosphate. Under aerobic conditions, it stores phosphate through the action of the polyphosphate kinase PPK, while under anaerobic conditions, it releases phosphate via the extracellular exopolyphosphatase PPX. The process of wastewater treatment typically involves utilizing the aforementioned characteristics of phosphorus-removing microorganisms to eliminate phosphorus from water [20]. The metabolic pathway of nitrogen and phosphorus removal by strain WD211 is shown in Figure 5. The relevant gene information is shown in Table S1.

#### 3.5.2. Genes Related to Organic Matter Degradation

The COD value, to a certain extent, indicates the content of organic matter in water. Figure 1a shows that strain WD211 exhibits a strong capability in removing organic matter. Aerobic bacteria possess numerous oxidative enzymes, such as oxygenase, which facilitate oxidation and play a crucial role in the degradation of organic substances [21,22]. Genomic analysis showed that a large number of genes encoding monooxygenase and dioxygenase were predicted in the genome of strain WD211. These included 37 genes encoding monooxygenase and 35 genes encoding dioxygenase (Table S2). Furthermore, RAST annotation revealed that strain WD211 possesses a significant number of genes involved in the degradation of aromatic compounds (Table S3). These genes included one quinic acid degradation gene, two biphenyl degradation genes, seven benzoate acid degradation genes, seven phenylpropanoic acid degradation genes, and three gentisate degradation genes.

### 3.6. Genomic Insight into Stress Resistance

#### 3.6.1. Genes Related to Antibiotic Resistance

Strain WD211 can grow on multiple antibiotic plates, indicating its possession of antibiotic resistance-related genes. The results predicted by the Comprehensive Antibiotic Research Database (CARD) show that the WD211 genome contains SoxR and AdeF genes. SoxR is a redox-sensitive transcriptional activator that induces the expression of a small regulon that includes the RND efflux pump-encoding operon mexGHI-opmD. SoxR was shown to be activated by pyocyanin. AdeF is the membrane fusion protein of the multidrug efflux complex AdeFGH. These results indicate that strain WD211 may acquire antibiotic resistance through the efflux of antibiotics. Furthermore, the genome annotation analysis revealed the presence of 19 genes associated with β-lactam resistance, 6 genes associated with vancomycin resistance, and 15 genes associated with cationic antimicrobial peptide (CAMP) resistance in the WD211 strain (Table S4). Antibiotics are typically organic compounds; therefore, genes associated with the degradation of organic compounds may also contribute to the acquisition of antibiotic resistance in strain WD211.

#### 3.6.2. Genes Related to Salt Resistance

Salt stress causes the accumulation of a large amount of Na^+^ inside the cell, which can lead to severe damage or even death. In this situation, the Na^+^/H^+^ antiporter plays a crucial role in maintaining normal salt concentrations inside the cell [23]. A Na^+^/H^+^ antiporter-encoding gene was found in the genome of strain WD211 (Table S5). It functions to excrete Na^+^ in a timely manner and maintain cell osmotic pressure.

Proline is one of the earliest osmoprotective agents found, with strong hydrophilicity and the highest solubility, which helps to prevent cell dehydration and relieve osmotic pressure [24]. The genes that are required for proline synthesis were found in the genome of strain WD211 (Table S5). These genes encode γ-glutamyl phosphate reductase, NADP-specific glutamate dehydrogenase, glutamate kinase, and pyrrolin-5-carboxylic acid reductase.

Betaine is also a common type of osmoprotectant, and its accumulation in bacteria involves synthesis from choline as a substrate and direct uptake from the external environment [25]. Genes related to betaine synthesis, encoded by choline dehydrogenase and betaine aldehyde dehydrogenase, were found in the WD211 genome. Betaine transport-related genes, encoded by OpuAA and OpuAC, are also present in this bacterial strain’s genome.

Trehalose is also an effective osmoprotectant [26]. The genome of strain WD211 harbours a total of five genes associated with the biosynthesis of trehalose. These genes, namely, *glgABC* and *TreYZ*, encode starch synthase, 1,4-α-glucan branching enzyme, glucose-1-phosphate adenylyltransferase, malto-oligosyltrehalose synthase, and malto-oligosyltrehalose trehalohydrolase. This information can be found in Table S5.

#### 3.6.3. Genes Related to the Two-Component System

**Figure 6:**
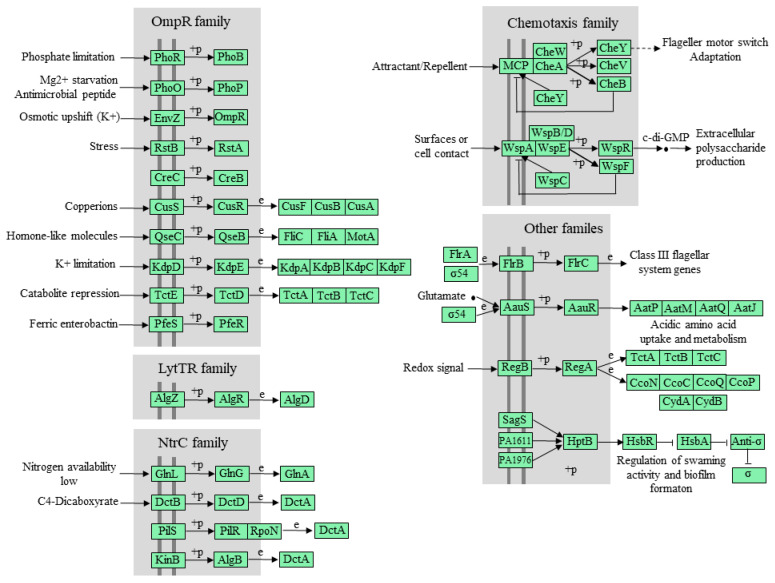
Two-component systems of strain WD211.

The two-component regulatory system is one of the ways in which bacteria adapt to their environment and is widely present in bacteria. This system consists of histidine kinase and a response regulator [27]. Through KEGG, the number and types of TCSs in strain WD211 were predicted. The results showed that there are 21 sets of TCSs in the genome of strain WD211 (Figure 6). Among them, GlnL/GlnG, PhoR/PhoB, KdpD/KdpE, and PhoQ/PhoP play important roles in responding to nitrogen, phosphorus, potassium, and magnesium limitations, respectively [28,29,30,31]. Studies have shown that PhoQ/PhoP can also improve the resistance of *P. aeruginosa* to polymyxin B by regulating an *arnBCADTEF*-*pmrE* operon that encodes lipopolysaccharide modification enzymes [32]. In addition, CusS/CusR also plays an important role in copper tolerance, silver tolerance, and antibiotic tolerance [33]. A large number of TCSs for strain WD211 were able to cope with complex environmental changes.

## 4. Discussion

*Pseudomonas* is one of the most numerous and complex bacterial genera. This genus of microorganisms exhibits a remarkable diversity of metabolic and physiological functions and is able to colonize different environments [34]. At present, many members of this genus have been proven to have the potential for application in many fields, such as plant growth promotion [35], plant disease prevention [36], and pollutant degradation [37]. Due to the diversity and complexity of the genus *Pseudomonas*, the taxonomic status of new isolates within the genus has been controversial since the genus was first described [11]. *P. chengduensis* was discovered in 2014, and phylogenetic analysis based on 16S rRNA could not effectively distinguish it from other *Pseudomonas* genera [14], indicating the complexity of its classification. At present, research on *P. chengduensis* is still very scarce, and the functional genes related to the purification of aquaculture sewage based on the complete genome have not been described. In this study, the whole genome of *P. chengduensis* WD211 isolated from fish ponds was sequenced using Illumina and OXfoad Nanopore sequencing techniques, and a high-quality complete genome of *P. chengduensis* WD211 was obtained. Based on the complete genome, we described the function of strain WD211 in the purification of aquaculture sewage. Meanwhile, we tested the effluent treatment capacity (nitrogen and phosphorus removal, organic matter degradation) as well as the resistance (salt tolerance, pH tolerance, antibiotic tolerance) of strain WD211.

Nitrogen in wastewater exists in either inorganic or organic form. Inorganic nitrogen mainly includes ammonia nitrogen and nitrate nitrogen [38], and organic nitrogen mainly includes proteins and amino acids [39]. After WD211 treatment, most of the ammonia nitrogen and nitrate nitrogen in the effluent had been removed, but the total nitrogen was only reduced by 18.12%. From the raw concentration of pollutants in the fish-pond effluent, it can be seen that the content of NH_4_^+^-N plus NO_3_^−^-N only accounted for 40% of the total nitrogen, which may be the reason for the large decrease in ammonia and nitrate nitrogen in the effluent, but the decrease in total nitrogen was smaller.

The salinity of mariculture wastewater is known to be approximately 3% [40]. Most of the current strains with the ability to remove nutrients from wastewater are not adapted to saline environments, resulting in significantly lower nutrient removal efficiencies [41,42,43]. Therefore, the application of salt-tolerant strains in mariculture wastewater treatment would be very beneficial for nutrient removal from saline effluents [40]. Strain WD211 was able to maintain a high level of biomass in NB medium containing 7% NaCl, which gives the strain the potential to remove nutrients from saline effluents, including mariculture wastewater. 

As aquaculture grows, an increasing number of antibiotics are being misused [44]. Although antibiotics are very effective in preventing and treating animal diseases, the large amount of antibiotics flowing into the environment can have a serious impact on environmental health by introducing antibiotic-resistant bacteria and killing aquatic microorganisms [45]. As an aquaculture sewage treatment strain, it is necessary to have a certain antibiotic resistance to play a purification role in antibiotic-containing sewage; otherwise, it will reduce the purification effect of sewage [9]. The antibiotic susceptibility test revealed that strain WD211 has developed resistance to multiple antibiotics, enabling it to be used in wastewater containing antibiotics. The antibiotic resistance of strain WD211 was mainly due to the presence of antibiotic-effector genes and numerous oxygenases in its genome. Oxygenase plays a key role in the degradation of organic matter [21]. The presence of large amounts of oxygenase enables the strain to remove large amounts of COD (Figure 1), while also potentially providing antibiotic resistance to the strain [46,47,48]. However, antibiotic resistance is a double-edged sword. On the one hand, it can make the strain stably colonize in the sewage containing antibiotics; on the other hand, horizontal gene transfer may accelerate the spread of antibiotic-resistant strains in the water environment. Therefore, such antibiotic-resistant microorganisms for treating aquaculture wastewater should preferably be immobilized rather than released directly into the water.

The whole genome of strain WD211 was sequenced to obtain a genome of 5,208,067 bp, encoding 4734 genes. The genome size of strain WD211 is smaller than that of the other two published complete genomes (BC18151 and T1624). By comparing the structure of the three genomes, it was found that the genome of strain WD211 was significantly different from the other two genomes. There are many locations in the genome where gene rearrangements occur (Figure 2). Gene rearrangement may introduce a new phenotype to strain WD211 [11]. In addition, 14 genomic islands were predicted in the genome of strain WD211. Genomic islands are considered horizontal gene transfer (HGT) events. The presence of gene islands often confers specific phenotypes on bacteria, such as antibiotic resistance and diverse metabolic adaptations [49,50,51].

The nitrogen metabolic pathway of strain WD211 showed that it lacked denitrification genes, and nitrogen in sewage was mainly removed through assimilation (Figure 3). However, it has been reported that the *P. chengduensis* strain BF6 could remove nitrogen through assimilation as it has a gene, *nirK,* encoding nitrite reductase, which reduces NO_2_^−^ to NO through denitrification [17]. The difference in nitrogen metabolism genes between strain WD211 and strain BF6 may be caused by different habitats. Strain BF6 was isolated from the biological filter, which was artificially supplemented with denitrifying bacteria. In the long-term evolution process, strain BF6 may have acquired denitrifying genes through horizontal gene transfer. In addition, the gene PPK for phosphorus accumulation and PPX for phosphorus release existed in the genome of strain WD211, and phosphorus removal could be achieved through the absorption and release of phosphorus under different conditions [20]. Moreover, strain WD211 has a large number of genes that help the strain improve environmental adaptability so that the strain can cope with the complex environmental stress factors in sewage to better perform water purification. However, the functional genes of strain WD211 are predicted based on the whole genome, and the specific gene functions need further experimental proof.

## 5. Conclusions

*P. chengduensis* WD211 was isolated from fish ponds. The sewage treated with strain WD211 showed a decrease in concentration of 18.12% in total nitrogen, 89.39% in NH_4_^+^, 62.16% in NO_3_^−^, 79.97% in total phosphorus, and 71.41% in COD after 24 h. Strain WD211 is able to grow in the pH range of 6–11 and can tolerate 7% NaCl as well as a variety of antibiotics. This demonstrates the high capacity of the strain to purify aquaculture sewage and its potential for application in effluents containing antibiotics or high salinity. The complete genome analysis showed that it had genes related to phosphorus and nitrogen removal, salt tolerance and antibiotic resistance, which provided a genetic basis for the development and utilization of its functions. These findings confirm that WD211 possesses excellent potential for application in aquaculture sewage remediation.

## Figures and Tables

**Table 1:** Test results of antibiotic resistance of strain WD211.

Antibiotic	Concentration	Inhibition Zone Diameter (mm)	Antibiotic	Concentration	Inhibition Zone Diameter (mm)
Penicillin	10 U/piece	0	Neomycin	30 μg/piece	0
Oxacillin	1 μg/piece	8	Tetracycline	30 μg/piece	0
Ampicillin	10 μg/piece	8	Doxycycline	30 μg/piece	0
Carbenicillin	100 μg/piece	0	Minocycline	30 μg/piece	0
Piperacillin	100 μg/piece	28	Erythrocin	15 μg/piece	0
Cefalexin	30 μg/piece	0	Medemycin	30 μg/piece	0
Cefazolin	30 μg/piece	0	Norfloxacin	10 μg/piece	16
Cefradine	30 μg/piece	0	Ofloxacin	5 μg/piece	23
Cefuroxime	30 μg/piece	8	Ciprofloxacin	5 μg/piece	15
Ceftazidime	30 μg/piece	0	Vancomycin	30 μg/piece	0
Ceftriaxone	30 μg/piece	0	Selectrin	23.75 μg/piece	0
Cefoperazone	75 μg/piece	0	Furazolidone	300 μg/piece	8
Amikacin	30 μg/piece	23	Chloramphenico	30 μg/piece	9
Gentamicin	30 μg/piece	0	Clindamycin	2 μg/piece	8
Kanamycin	30 μg/piece	0			

**Table 2:** General genome features of strain WD211.

Features	Chromosome
Genome total size(bp)	5,208,067
G + C content	62.75%
Coding genes	4734
Plasmid	0
rRNAs	12
tRNAs	67
Pseudogene	1
CRISPRs	2
Genomic islands	14

## Data Availability

Data are available from the corresponding authors upon reasonable request.

## Supplementary Materials

The following supporting information can be downloaded at: https://www.mdpi.com/article/10.3390/genes14122107/s1.

## References

[1] Huang Y., Ciais P., Goll D.S., Sardans J., Peñuelas J., Cresto-Aleina F., Zhang H. (2020). The shift of phosphorus transfers in global fisheries and aquaculture. Nat. Commun..

[2] Hu Z., Lee J.W., Chandran K., Kim S., Khanal S.K. (2012). Nitrous oxide (N_2_O) emission from aquaculture: A review. Environ. Sci. Technol..

[3] van Rijn J. (2013). Waste treatment in recirculating aquaculture systems. Aquac. Eng..

[4] Chai Y., Peng R., Jiang M., Jiang X., Han Q., Han Z. (2022). Effects of ammonia nitrogen stress on the blood cell immunity and liver antioxidant function of Sepia pharaonis. Aquaculture.

[5] Zainab S.M., Junaid M., Xu N., Malik R.N. (2020). Antibiotics and antibiotic resistant genes (ARGs) in groundwater: A global review on dissemination, sources, interactions, environmental and human health risks. Water Res..

[6] Liang D.H., Hu Y.Y., Cheng J.H., Chen Y.C. (2020). Simultaneous sulfamethoxazole biodegradation and nitrogen conversion in low C/N ratio pharmaceutical wastewater by Achromobacter sp. JL9. Sci. Total Environ..

[7] Jin P., Chen Y.Y., Xu T., Cui Z.W., Zheng Z.W. (2019). Efficient nitrogen removal by simultaneous heterotrophic nitrifying-aerobic denitrifying bacterium in a purification tank bioreactor amended with two-stage dissolved oxygen control. Bioresour. Technol..

[8] Chen J., Sun R., Pan C., Sun Y., Mai B., Li Q.X. (2020). Antibiotics and Food Safety in Aquaculture. J. Agric. Food Chem..

[9] Wu L., Wei Q., Zhang Y., Fan Y., Li M., Rong L., Xiao X., Huang X., Zou X. (2021). Effects of antibiotics on enhanced biological phosphorus removal and its mechanisms. Sci. Total Environ..

[10] Peix A., Ramírez-Bahena M.H., Velázquez E. (2018). The current status on the taxonomy of Pseudomonas revisited: An update. Infect. Genet. Evol..

[11] Silby M.W., Winstanley C., Godfrey S.A., Levy S.B., Jackson R.W. (2011). Pseudomonas genomes: Diverse and adaptable. FEMS Microbiol. Rev..

[12] Saati-Santamaría Z., Baroncelli R., Rivas R., García-Fraile P. (2022). Comparative Genomics of the Genus Pseudomonas Reveals Host- and Environment-Specific Evolution. Microbiol. Spectr..

[13] Kchouk M., Gibrat J.F., Elloumi M. (2017). Generations of sequencing technologies: From first to next generation. Biol. Med..

[14] Tao Y., Zhou Y., He X., Hu X., Li D. (2014). Pseudomonas chengduensis sp. nov., isolated from landfill leachate. Int. J. Syst. Evol. Microbiol..

[15] Shi Z., Yu X.L., Duan J., Guo W. (2023). The complete genome sequence of Pseudomonas chengduensis BC1815 for genome mining of PET degrading enzymes. Mar. Genom..

[16] Wang X., Li D., Gao P., Gu W., He X., Yang W., Tang W. (2020). Analysis of biosorption and biotransformation mechanism of Pseudomonas chengduensis strain MBR under Cd(II) stress from genomic perspective. Ecotoxicol. Environ. Saf..

[17] Yi M., Wang H., Ma X., Wang C., Wang M., Liu Z., Lu M., Cao J., Ke X. (2023). Efficient nitrogen removal of a novel Pseudomonas chengduensis strain BF6 mainly through assimilation in the recirculating aquaculture systems. Bioresour. Technol..

[18] State Environmental Protection Administration (2002). Methods for Monitoring and Analysis of Water and Wastewater.

[19] Darling A.E., Tritt A., Eisen J.A., Facciotti M.T. (2011). Mauve assembly metrics. Bioinformatics.

[20] Dai H., Sun Y., Wan D., Abbasi H.N., Guo Z., Geng H., Wang X., Chen Y. (2022). Simultaneous denitrification and phosphorus removal: A review on the functional strains and activated sludge processes. Sci. Total Environ..

[21] Kumari S., Das S. (2023). Bacterial enzymatic degradation of recalcitrant organic pollutants: Catabolic pathways and genetic regulations. Environ. Sci. Pollut. Res. Int..

[22] Gangola S., Joshi S., Kumar S., Pandey S.C., Bhatt P. (2019). Comparative analysis of fungal and bacterial enzymes in biodegradation of xenobiotic compounds. Smart Bioremediation Technologies.

[23] Padan E., Schuldiner S. (1994). Molecular physiology of the Na^+^/H^+^ antiporter in *Escherichia coli*. J. Exp. Biol..

[24] Li J., Wu H., Pu Q., Zhang C., Chen Y., Lin Z., Hu X., Li O. (2023). Complete genome of Sphingomonas paucimobilis ZJSH1, an endophytic bacterium from Dendrobium officinale with stress resistance and growth promotion potential. Arch. Microbiol..

[25] Cayley S., Lewis B.A., Record M.T. (1992). Origins of the osmoprotective properties of betaine and proline in Escherichia coli K-12. J. Bacteriol..

[26] Iturriaga G., Suárez R., Nova-Franco B. (2009). Trehalose metabolism: From osmoprotection to signaling. Int. J. Mol. Sci..

[27] Tierney A.R., Rather P.N. (2019). Roles of two-component regulatory systems in antibiotic resistance. Future Microbiol..

[28] Pahel G., Rothstein D.M., Magasanik B. (1982). Complex glnA-glnL-glnG operon of *Escherichia coli*. J. Bacteriol..

[29] Baek J.H., Kang Y.J., Lee S.Y. (2007). Transcript and protein level analyses of the interactions among PhoB, PhoR, PhoU and CreC in response to phosphate starvation in *Escherichia coli*. FEMS Microbiol. Lett..

[30] Heermann R., Jung K. (2010). The complexity of the ‘simple’ two-component system KdpD/KdpE in *Escherichia coli*. FEMS Microbiol. Lett..

[31] Groisman E.A., Duprey A., Choi J. (2021). How the PhoP/PhoQ System Controls Virulence and Mg^2+^ Homeostasis: Lessons in Signal Transduction, Pathogenesis, Physiology, and Evolution. Microbiol. Mol. Biol. Rev..

[32] Yang B., Liu C., Pan X., Fu W., Fan Z., Jin Y., Bai F., Cheng Z., Wu W. (2021). Identification of Novel PhoP-PhoQ Regulated Genes That Contribute to Polymyxin B Tolerance in Pseudomonas aeruginosa. Microorganisms.

[33] Chen D., Zhao Y., Qiu Y., Xiao L., He H., Zheng D., Li X., Yu X., Xu N., Hu X. (2020). CusS-CusR Two-Component System Mediates Tigecycline Resistance in Carbapenem-Resistant Klebsiella pneumoniae. Front. Microbiol..

[34] Hesse C., Schulz F., Bull C.T., Shaffer B.T., Yan Q., Shapiro N., Hassan K.A., Varghese N., Elbourne L.D.H., Paulsen I.T. (2018). Genome-based evolutionary history of *Pseudomonas* spp.. Environ. Microbiol..

[35] Li Q., Li H., Yang Z., Cheng X., Zhao Y., Qin L., Bisseling T., Cao Q., Willemsen V. (2022). Plant growth-promoting rhizobacterium Pseudomonas sp. CM11 specifically induces lateral roots. New Phytol..

[36] Khare E., Arora N.K. (2021). Biosurfactant based formulation of Pseudomonas guariconensis LE3 with multifarious plant growth promoting traits controls charcoal rot disease in Helianthus annus. World J. Microbiol. Biotechnol..

[37] Yesankar P.J., Patil A., Kapley A., Qureshi A. (2023). Catalytic resilience of multicomponent aromatic ring-hydroxylating dioxygenases in Pseudomonas for degradation of polycyclic aromatic hydrocarbons. World J. Microbiol. Biotechnol..

[38] Li Z., Li L., Sun H., Wang W., Yang Y., Qi Z., Liu X. (2022). Ammonia assimilation: A double-edged sword influencing denitrification of Rhodobacter azotoformans and for nitrogen removal of aquaculture wastewater. Bioresour. Technol..

[39] Zheng F., Wang J., Xiao R., Chai W., Xing D., Lu H. (2021). Dissolved organic nitrogen in wastewater treatment processes: Transformation, biosynthesis and ecological impacts. Environ. Pollut..

[40] Zhang M., Pan L., Su C., Liu L., Dou L. (2021). Simultaneous aerobic removal of phosphorus and nitrogen by a novel salt-tolerant phosphate-accumulating organism and the application potential in treatment of domestic sewage and aquaculture sewage. Sci. Total Environ..

[41] Zhu B.T., Chen S.C., Zhao C.G., Zhong W.H., Zeng R.Y., Yang S.P. (2019). Effects of Marichromatium gracile YL28 on the nitrogen management in the aquaculture pond water. Bioresour. Technol..

[42] Uygur A., Kargi F. (2004). Salt inhibition on biological nutrient removal from saline wastewater in a sequencing batch reactor. Enzym. Microb. Technol..

[43] Intrasungkha N., Keller J., Blackall L.L. (1999). Biological nutrient removal efficiency in treatment of saline wastewater. Water Sci. Technol..

[44] Adenaya A., Berger M., Brinkhoff T., Ribas-Ribas M., Wurl O. (2023). Usage of antibiotics in aquaculture and the impact on coastal waters. Mar. Pollut. Bull..

[45] Hatosy S.M., Martiny A.C. (2015). The Ocean as a Global Reservoir of Antibiotic Resistance Genes. Appl. Environ. Microbiol..

[46] Minerdi D., Zgrablic I., Castrignanò S., Catucci G., Medana C., Terlizzi M.E., Gribaudo G., Gilardi G., Sadeghi S.J. (2015). *Escherichia coli* Overexpressing a Baeyer-Villiger Monooxygenase from Acinetobacter radioresistens Becomes Resistant to Imipenem. Antimicrob. Agents Chemother..

[47] Liu L.K., Abdelwahab H., Del Campo J.S.M., Mehra-Chaudhary R., Sobrado P., Tanner J.J. (2016). The Structure of the Antibiotic Deactivating, N-hydroxylating Rifampicin Monooxygenase. J. Biol. Chem..

[48] dos Santos D.F., Istvan P., Noronha E.F., Quirino B.F., Krüger R.H. (2015). New dioxygenase from metagenomic library from Brazilian soil: Insights into antibiotic resistance and bioremediation. Biotechnol. Lett..

[49] Battle S.E., Rello J., Hauser A.R. (2009). Genomic islands of Pseudomonas aeruginosa. FEMS Microbiol. Lett..

[50] Bellanger X., Payot S., Leblond-Bourget N., Guédon G. (2014). Conjugative and mobilizable genomic islands in bacteria: Evolution and diversity. FEMS Microbiol. Rev..

[51] Hall R.M. (2010). Salmonella genomic islands and antibiotic resistance in Salmonella enterica. Future Microbiol..

