# Supplementary material for: Exploring the Application Potential of Aquaculture Sewage Treatment of Pseudomonas chengduensis Strain WD211 Based on Its Complete Genome
Source: Genes (Basel). 2023 Nov 21;14(12):2107. doi: 10.3390/genes14122107 (PMC10743257; doi:10.3390/genes14122107)
Supplement: Supplementary file 1 [file genes-14-02107-s001.zip › genes-2678446-supplementary.pdf]

Table S1. Genes related to nitrogen and phosphorus removal

Gene ID	Product	Description
GE002048/GE002049/GE004027 /GE004028	Nitrate/nitrite transport system substrate-binding protein nrtA/B	Nitrogen removal
GE002050/GE004029	Nitrate/nitrite transport system ATP-binding protein nrtC/D	
GE000257	Assimilatory nitrate reductase catalytic subunit nasA	
GE004037	Assimilatory nitrate reductase electron transfer subunit nasB	
GE004035	Nitrite reductase (NADH) large subunit nirB	
GE004036	Nitrite reductase (NADH) small subunit nirD	
GE000240/GE001777/GE004623	Ammonium transporter	
GE001424/GE001862/GE001863 /GE002184/GE003535	Glutamine synthetase glnA	
GE002045	Glutamate synthase (NADPH) large subunit gltB	
GE002046	Glutamate synthase (NADPH) small subunit gltD	
GE000160/GE004499/GE004538	Glutamate dehydrogenase gluD	
GE003306	Glutamate dehydrogenase (NADP+) gdhA	
GE001677	Phosphate transport system permease protein PstA	Phosphorus removal
GE001678	Phosphate transport system ATP-binding protein PstB	
GE001676	Phosphate transport system permease protein PstC	
GE001675/ GE003662	Phosphate transport system substrate-binding protein PstS	
GE001822/ GE000141	Polyphosphate kinase PPK	
GE001823	Exopolyphosphatase PPX	

Table S2. Genes related to organic matter degradation

Gene ID	Product	Description
GE001847/GE001848/GE002949/ GE003521/GE003758/GE004200/ GE004724/GE003244	Monooxygenase activity	Monooxygenase
GE001895	FAD-containing monooxygenase EthA	
GE000027	Phenylalanine 4-monooxygenase	
GE000068/ GE000201/ GE000621/ GE001263/ GE002655/ GE003236/ GE003500/ GE003532/ GE004208	Antibiotic biosynthesis monooxygenase	
GE000762	P-hydroxybenzoate 3-monooxygenase	
GE000888/ GE002063	nitronate monooxygenase	
GE000894	2-polyprenyl-3-methyl-6-methoxy-1,4-benzoquinone monooxygenase	
GE001028	Carnitine monooxygenase oxygenase subunit	
GE001225	Alkanesulfonate monooxygenase	
GE001243/ GE004201	Probable FMNH2-dependent monooxygenase SfnC	
GE001355	Luciferase-like monooxygenase	
GE003047	FAD-binding monooxygenase	
GE003236/ GE004208	Heme-degrading monooxygenase HmoA	
GE003549	Cyclohexanone monooxygenase	
GE003834	N, N-dimethylaniline monooxygenase	
GE004202	Dimethyl-sulfide monooxygenase	
GE004350	4-hydroxyacetophenone monooxygenase	
GE004372	Alkane 1-monooxygenase	
GE004496	L-ornithine N5-monooxygenase	
GE000033/ GE000648/ GE001111/ GE001413/ GE003243/ GE004009/ GE004012/ GE004342	Dioxygenase activity	Dioxygenase
GE000119/ GE003522	Phytanoyl-CoA dioxygenase	
GE000445	Nitric oxide dioxygenase	
GE000481	Ring-hydroxylating dioxygenase	
GE000753/ GE003237	4-hydroxyphenylpyruvate dioxygenase	
GE000777	Catechol 1,2-dioxygenase	
GE000783	Benzoate 1,2-dioxygenase small subunit	
GE000784	Benzoate 1,2-dioxygenase large subunit	
GE000789	Protocatechuate 3,4-dioxygenase alpha chain	
GE000790/ GE001970	Protocatechuate 3,4-dioxygenase beta chain	
GE000888/ GE002063	2-Nitropropane dioxygenase	
GE001554	DOPA 4,5-dioxygenase	
GE002176/ GE002784	Nitric oxide dioxygenase	
GE002329	Fe^{2+}-dependent dioxygenase	
GE002728/ GE003076/ GE003614	Quercetin 2,3-dioxygenase	
GE002982	Alpha-ketoglutarate-dependent dioxygenase AlkB	
GE003238	Homogentisate 1,2-dioxygenase	
GE004116	4,5-DOPA dioxygenase extradiol	
GE004216	Alpha-ketoglutarate-dependent taurine dioxygenase	
GE004356	Acireductone dioxygenase	

GE004488	Taurine catabolism dioxygenase

Table S3. Genes related to aromatic compounds degradation

Gene ID	Product	Description
GE000780	Benzoate MFS transporter BenK	Benzoate degradation
GE000784	Benzoate 1,2-dioxygenase large subunit	
GE000782	Benzoate 1,2-dioxygenase, ferredoxin reductase component	
GE000783	Benzoate 1,2-dioxygenase small subunit	
GE000781	1,2-dihydroxycyclohexa-3,5-diene-1-carboxylate dehydrogenase	
GE000776/ GE003485	Benzoate transport protein	
GE000785	benABC operon transcriptional activator BenR	
GE000140/ GE000559/ GE002262/ GE003086/ GE003260/ GE003576/ GE004162	3-ketoacyl-CoA thiolase	n-Phenylalkanoic acid degradation
GE000169/ GE002991	Enoyl-CoA hydratase	
GE000181/ GE000420/ GE002993/ GE002994/ GE003259/ GE003515/ GE003631/ GE004599/	Long-chain-fatty-acid--CoA ligase	
GE001900/ GE003085/ GE003577	3-hydroxybutyryl-CoA epimerase	
GE004009	Biphenyl-2,3-diol 1,2-dioxygenase III-related protein	Biphenyl Degradation
GE002636	2-hydroxy-6-oxo-6-phenylhexa-2, 4-dienoate hydrolase	
GE000750/ GE002235	3-dehydroquinate dehydratase II	Quinate degradation
GE003240	Maleylacetoacetate isomerase	Gentisate degradation
GE001903	Putative 4-hydroxybenzoyl-CoA thioesterase	
GE003516	Fumarylacetoacetate hydrolase family protein	

Table S4. Genes related to antibiotic resistance

Gene ID	Product	Description
GE003095	Beta-N-acetylhexosaminidase	Beta-Lactam resistance
GE004160	Beta-lactamase class C	
GE000625/ GE002349/ GE004319	Membrane fusion protein	
GE002446	Cell division protein FtsI	
GE002036	Penicillin-binding protein 1A	
GE00071/ GE002446	Penicillin-binding protein 2	
GE002471	Beta-lactamase induction signal transducer AmpG	
GE002107	Outer membrane protein	
GE000970/ GE002598/ GE002751	Imipenem/basic amino acid-specific outer membrane pore	
GE002348	Repressor of the mexAB-oprM multidrug resistance operon	
GE000626/ GE002350/GE004318	Multidrug efflux pump	
GE002107	Outer membrane protein	
GE002449	Phospho-N-acetylmuramoyl-pentapeptide-transferase	Vancomycin resistance
GE001763/ GE002147	Alanine racemase	
GE002454	D-alanine-D-alanine ligase	
GE002448	UDP-N-acetylmuramoyl-tripeptide--D-alanyl-D-alanine ligase	
GE002452	UDP-N-acetylglucosamine--N-acetylmuramyl-(pentapeptide) pyrophosphoryl-undecaprenol N-acetylglucosamine transferase	
GE004668	UDP-N-acetylglucosamine acyltransferase	Cationic antimicrobial peptide (CAMP) resistance
GE002129	N-acetylmuramoyl-L-alanine amidase	
GE000625/ GE002349/ GE004319	Membrane fusion protein	
GE001656	Protein dithiol oxidoreductase	
GE002785	Peptidyl-prolyl cis-trans isomerase A	
GE002966	Serine protease Do	
GE004605	OmpR family, sensor histidine kinase PhoQ	
GE004606	OmpR family, response regulator PhoP	
GE002107	Outer membrane protein	
GE001527	Phosphatidylglycerol lysyltransferase	
GE000626/ GE002350/ GE004318	Multidrug efflux pump	

Table S5. Genes related to osmotic resistance

Gene ID	Product	Description
GE000387	Na^+ /H^+ antiporter NhaC	Na^+ transport
GE000388	Na^+ /H^+ antiporter NhaD	
GE002918	Na^+ /H^+ antiporter subunit G	
GE002919	Na^+ /H^+ antiporter subunit F	
GE002920/ GE002938	Na^+ /H^+ antiporter subunit E	
GE002921	Na^+ /H^+ antiporter subunit D	
GE002922	Na^+ /H^+ antiporter subunit C	
GE002923	Na^+ /H^+ antiporter subunit A	
GE003128	Na^+ /H^+ antiporter NhaA	
GE001042	Pyrroline-5-carboxylate reductase	Proline synthesis
GE000577	Glutamate 5-kinase	
GE003306	NADP-specific glutamate dehydrogenase	
GE000715	Gamma-glutamyl phosphate reductase	
GE001978	Choline dehydrogenase	Betaine synthesis and transport
GE001979	Betaine aldehyde dehydrogenase	
GE001984	Glycine betaine ABC transport system, ATP-binding protein OpuAA	
GE001982	Glycine betaine ABC transport system, glycine betaine-binding protein OpuAC	
GE003978	Malto-oligosyltrehalose trehalohydrolase	Trehalose synthesis
GE003994	1,4-alpha-glucan (glycogen) branching enzyme	
GE003980	Malto-oligosyltrehalose synthase	
GE003996	Alpha-amylase	
GE003995	Trehalose synthase	
